# Modeling of pressure–volume controlled artificial respiration with local derivatives

**DOI:** 10.1186/s13662-020-03204-9

**Published:** 2021-01-14

**Authors:** Bahar Acay, Mustafa Inc, Yu-Ming Chu, Bandar Almohsen

**Affiliations:** 1 Department of Mathematics, Firat University, Elazig, Turkey; 2 Department of Medical Research, China Medical University, Taichung, Taiwan; 3 Department of Mathematics, Huzhou University, Huzhou, China; 4 Hunan Provincial Key Laboratory of Mathematical Modeling and Analysis in Engineering, Changsha University of Science & Technology, Changsha, China; 5 Department of Mathematics, King Saud University, Riyadh, Saudi Arabia

**Keywords:** Proportional derivative, Local derivatives, Clinical medicine, Truncated M-derivative, Conformable derivative

## Abstract

We attempt to motivate utilization of some local derivatives of arbitrary orders in clinical medicine. For this purpose, we provide two efficient solution methods for various problems that occur in nature by employing the local proportional derivative defined by the proportional derivative (PD) controller. Under some necessary assumptions, a detailed exposition of the instantaneous volume in a lung is furnished by conformable derivative and such modified conformable derivatives as truncated *M*-derivative and proportional derivative. Moreover, we wish to investigate the performance of the above-mentioned operators in applications by plotting several graphs of the governing equations.

## Introduction

In medicine, mechanical ventilation (assisted ventilation) is a supportive treatment provided by a medical machine named a ventilator. This breathing machine is utilized for severe illnesses in an intensive care unit (ICU) in case of breathing failure, coma, neuromuscular disorders, acute severe asthma, and so on. It is also used to get rid of carbon dioxide to supply oxygen into the lungs, to facilitate breathing, or to breathe for critically ill patients. Differently from the many specific types of mechanical ventilation, there are two main mechanical ventilations involving positive pressure ventilation and negative pressure ventilation. The former pushes air or gas into the lungs, and the latter sucks air into the lungs by stimulating chest movement. The ventilator is connected to the patient by a tube in windpipe through the nose or mouth and blows air plus oxygen needed into the patient’s lung. Also, positive end-expiratory pressure (PEEP) can be provided by a ventilator, which helps to hold the lungs open to prevent the air sacs from collapsing. Patients on a ventilator providing more oxygen than other devices like masks are monitored to control the respiratory rate, heart rate, oxygen saturation, and blood pressure. Besides the benefits of using a ventilator, there are also some risks. The ventilator itself is not a method of treatment, it only ensures support until the patient feels better or heals. Moreover, people on ventilators cannot talk or eat, and some are uncomfortable with a tube (endotracheal or ET tube) in their nose or mouth. It can cause an infection like pneumonia because the tube allows bacteria to easily get into the person’s lung. Occasionally, the lung may collapse owing to getting full of air, and in addition to this, lung damage, side effects of medications, inability to discontinue ventilator support, and alveolar damage can be regarded among the risks of the ventilator. Hence the health care team all the time tries to help a patient get rid of the ventilator as soon as possible.

This study is intended to observe the model of the mechanical process of a ventilator as appeared in [1]. Some assumptions must be made for this process of filling the lungs with air and letting them deflate to some volume. The lung is modeled by a single compartment. The ventilator applies a constant pressure $P_{d}$ to the airway, and it is zero during expiration. Each breath length is fixed by $t_{b}$ determined by the clinician, and $t_{j}$ denotes the inspiratory time. The pressure of the ventilator is denoted by $P_{d}$. Additionally, the pressure balance at the airway is presented by

1 $$ P_{l}+P_{k}+P_{m}=P_{aw}, $$

where $P_{l}$ stands for airway-resistance drop, $P_{k}$ is the lung elastic pressure, $P_{m}$ is the residual pressure, and the pressure applied to airway is denoted by $P_{aw}$. In addition, $P_{m}$ can be computed by the condition $\mathcal{V}_{e}(t_{b})=0$ as given in the following formula:

2 $$ P_{m}=\frac{(e^{t_{j}/RC}-1)P_{d}}{e^{t_{b}/RC}-1}. $$

Furthermore, the mean alveolar pressure, which is the average pressure in the lung during inspiration, is calculated by the condition $\mathcal{V}_{i}(0)=0$ as follows:

3 $$ P_{ma}=\frac{1}{Ct_{j}} \int _{0}^{t_{j}}\mathcal{V}_{i}(t) \,dt+P_{m}. $$

Under the assumptions above and by utilizing the pressure equation (1), a model for the instantaneous volume in a lung is presented by

4 $$\begin{aligned} &R \biggl(\frac{d\mathcal{V}_{i}(t)}{dt} \biggr)+ \biggl(\frac{1}{C} \biggr) \mathcal{V}_{i}(t)+P_{m}=P_{d}, \quad 0\le t\le t_{j}, \end{aligned}$$

5 $$\begin{aligned} &R \biggl(\frac{d\mathcal{V}_{e}(t)}{dt} \biggr)+ \biggl(\frac{1}{C} \biggr) \mathcal{V}_{e}(t)+P_{m}=0, \quad t_{j}\le t\le t_{b}, \end{aligned}$$

6 $$\begin{aligned} &\mathcal{V}_{i}(0)=\mathcal{V}_{e}(t_{b})=0, \end{aligned}$$

7 $$\begin{aligned} &\mathcal{V}_{i}(t_{j})=\mathcal{V}_{e}(t_{j})= \mathcal{V}_{T}, \end{aligned}$$

where $\mathcal{V}_{i}(t)$ is the volume during inspiration, $\mathcal{V}_{e}(t)$ denotes the volume during expiration, and $\mathcal{V}_{T}$ stands for the tidal volume of the breath. It is assumed that $P_{l}$ is proportional to the flows into and out of the lung and $P_{k}$ is proportional to the instantaneous volume of the lung; that is, $P_{l}=R (\frac{d\mathcal{V}(t)}{dt} )$ and $P_{k}= (\frac{1}{C} )\mathcal{V}(t)$, where *C* is a constant called the compliance of the lung.

In today’s world, fractional calculus has made a big impression in various scientific study fields like mathematics, physics, engineering, psychology, biology, and so on. With many advantageous results, as predicted by Leibniz, noninteger orders of derivative and integral are utilized to model real-world problems in the above-mentioned venerable fields. Using fractional operators is a novel modeling perspective especially on mathematics, which enables us to observe key points of the model and to find various solutions thanks to different types of fractional derivatives. One of these definitions, probably the most important and general one, is that of Riemann–Liouville created through a complex analysis approach. This leading fractional integral and derivative definition with the power-function kernel is defined by

8 $$\begin{aligned} &{} _{ a}^{RL}\mathbf{I}^{\alpha }\psi (t)= \frac{1}{\Gamma (\alpha )} \int _{a}^{t}(t-\tau )^{\alpha -1}\psi ( \tau )\,d \tau, \end{aligned}$$

9 $$\begin{aligned} &{}_{ a}^{RL}\mathbf{D}^{\alpha }\psi (t)={ \frac{d^{n}}{dt^{n}}} {_{ a}^{RL}\mathbf{I}^{n-\alpha } \psi (t)}, \end{aligned}$$

where $\operatorname{Re}(\alpha )>0$ in (8), $\operatorname{Re}(\alpha )\ge 0$ in (9), and $n=\lfloor \operatorname{Re}(\alpha )\rfloor +1$. Unfortunately, it is not enough to describe problems only concerning power-law behavior because there are various applications in nature, which may not be described by a basic power function. For this reason, many authors have alternatively furnished fractional operators having different types of kernels. To see a good deal of definitions containing varied kernels, we refer the reader to [2–4], and for some beneficial comments on creating different fractional operators, we refer the reader to [5]. One of the main reasons for the desire to introduce novel fractional operators or generalizations of already existing operators is expanding and diversifying the underlying field. In doing so, however, questions arise as to which operator matches the criteria of fractional derivative and integral definition. Although there are no clear and precise criteria whether it does, following the definition of fractional derivatives, there are two separate classes of operators, local and nonlocal, in the literature. Whereas nonlocal operators have memory effect, seen as an advantage, local ones, limit-based definitions, have no memory-effect. Nonlocal derivatives are more useful, but it is well known that local derivatives are a vital tool for obtaining nonlocal derivatives. As a substantial example of local derivative, we can give the conformable derivative introduced by Khalil et al. [6] as follows:

10 $$ _{C}\mathbf{D}^{\alpha }\psi (t)=\lim _{\varepsilon \rightarrow 0} \frac{\psi (t+\varepsilon t^{1-\alpha })-\psi (t)}{\varepsilon }, $$

where $\psi:[0,\infty )\rightarrow \mathbb{R}$ and $0<\alpha <1$. After this popular local derivative definition, many authors introduced several modified conformable derivatives for *α*-differentiable functions. Replacing $\varepsilon t^{1-\alpha }$ in (10) by $te^{\varepsilon t^{-\alpha }}$, Katugampola [7] presented another limit-based derivative, and then by adding the Mittag-Leffler function instead of the exponential function in Katugampola definition, Sousa et al. [8] put a more general local derivative forward. Moreover, inserting $(t+\frac{1}{\Gamma (\alpha )} )^{1-\alpha }$ into the limit definition, Atangana [9] provided a different type of conformable derivative to solve a partial differential equation. All these local derivatives are useful mathematical tools, which are compatible with many theorems and properties in classical analysis and contain arbitrary order. For a deeper discussion and information about conformable derivative and other counterparts, see [10, 12–14] and references therein. In addition to the advantages of these limit-based local derivatives, they also have some shortcomings; for example, the identity operator is not obtained as $\alpha \rightarrow 1$, that is, ${}_{C}\mathbf{D}^{0}\psi (t)\ne \psi (t)$, and the variable *t* in (10) must satisfy the condition $t\ge 0$. To complement these deficiencies, Anderson and Ulness [15] offered a novel local derivative definition for $\alpha \in [0,1]$ and $t\in \mathbb{R}$, where ${}_{P}\mathbf{D}^{0}\psi (t)=\psi (t)$. When describing this remarkable derivative, they used the proportional derivative (PD) controller and provided a useful derivative definition with its corresponding integral. To learn more about proportional-integral-derivative (PID) control, which provides an efficient solution to real-world problems including the integral and derivative terms, the best general reference is [16]. After all these limit-based local derivatives are introduced, many authors performed their nonlocal cases we mentioned as fractional by benefiting from the idea of creating Riemann–Liouville definition (8). Accordingly, by iterating the corresponding integral of a local derivative, a fractional operator having a memory effect can be obtained. See [17–35] for more detail about such fractional operators.

This study is created as follows. In Sect. 2, we first set up notation and terminology to present fundamental concepts of some different types of local derivatives such as proportional derivative, truncated *M*-derivative, and conformable derivative. Section 3 is devoted to giving two crucial methods to solve a great number of differential equations. We introduce the proportional variation-of-parameters method and proportional Laplace transform (LT-p). So we touch some aspects of the theory of proportional derivatives. Additionally, in this section, we present the solution of the mass-spring system employing proportional variation-of-parameter method as an application. Furthermore, in Sect. 4, we give a model in clinical medicine showing the instantaneous volume in a lung as an application of LT-p. This important model is also solved by truncated *M*-derivative and conformable derivative to compare with each other. Lastly, discussion and conclusions on obtained results are exhibited by plotting various graphs for both equations of the lung volume during inspiration and during expiration.

## Fundamental concepts of some local derivatives

In this section, we present some important definitions and theorems about proportional derivative, truncated *M*-derivative, and conformable derivative necessary for the main results of this study.

### Definition 2.1

([15])

A proportional derivative controller for $u(t)$ defined as the controller output with two tuning parameters $\kappa _{p}$ and $\kappa _{d}$ is

11 $$ u(t)=\kappa _{p}{e(t)}+\kappa _{d} \frac{de(t)}{dt}, $$

where *t* is the time or instantaneous time, $e(t)$ is the error, $\kappa _{p}$ is the proportional gain, and $\kappa _{d}$ is the derivative gain.

### Definition 2.2

([15])

Let $0\le \alpha \le 1$, and let $\kappa _{0},\kappa _{1}:[0,1]\times \mathbb{R}\rightarrow [0,\infty )$ be continuous functions with the following properties:

12 $$\begin{aligned} &\lim_{\alpha \rightarrow 0^{+}}\kappa _{1}(\alpha,t)=1,\qquad \lim_{\alpha \rightarrow 0^{+}}\kappa _{0}(\alpha,t)=0, \end{aligned}$$

13 $$\begin{aligned} & \lim_{\alpha \rightarrow 1^{-}}\kappa _{1}(\alpha,t)=1,\qquad \lim_{\alpha \rightarrow 1^{-}}\kappa _{0}(\alpha,t)=1, \end{aligned}$$

and $\kappa _{1}(\alpha,t)\ne 0, 0\le \alpha <1$, $\kappa _{0}(\alpha,t)\ne 0, 0<\alpha \le 1$, for all $t \in \mathbb{R}$.

Then the proportional derivative of order *α* is defined as

14 $$ _{P}\mathbf{D}^{\alpha }\phi (t)=\kappa _{1}(\alpha,t)\phi (t)+\kappa _{0}( \alpha,t)\phi '(t). $$

Especially, as done in [11], replacing $\kappa _{1}(\alpha,t)$ by $(1-\alpha )$ and $\kappa _{0}(\alpha,t)$ by *α*, as an alternative to (14), we can use the following definition:

15 $$ _{P}\mathbf{D}^{\alpha }\phi (t)=(1-\alpha )\phi (t)+\alpha \phi '(t), $$

and the corresponding proportional integral is defined by

16 $$ _{P}\mathbf{I}^{\alpha }\phi (t)= \int _{a}^{t} e^{ \frac{\alpha -1}{\alpha }(t-\tau )}\phi (\tau ) \,d_{\alpha }\tau,\quad d_{\alpha }\tau =\frac{1}{\alpha }\,d\tau. $$

### Definition 2.3

([15] Proportional exponential function)

Let $0<\alpha \le 1$, let $r,t\in \mathbb{R}$ be such that $r\le t$, and let $\Theta:[r,t]\rightarrow \mathbb{R}$ be a continuous function. Also, let $\kappa _{0}(\alpha,t)$ and $\kappa _{1}(\alpha,t)$ satisfy (12)–(13). Then the proportional exponential function is given by

17 $$ e_{\Theta }(t,r)=e^{-\int _{r}^{t} \frac{\kappa _{1}(\alpha,\tau )-\Theta (\tau )}{\kappa _{0}(\alpha,\tau )}\,d \tau }, $$

and for $\Theta =0$, we can use the proportional exponential function

18 $$ e_{0}(t,r)=e^{-\int _{r}^{t} \frac{\kappa _{1}(\alpha,t)}{\kappa _{0}(\alpha,t)}\,d\tau }. $$

### Definition 2.4

([15])

Let $0<\alpha \le 1$, let the functions $\kappa _{1}(\alpha,t)$ and $\kappa _{0}(\alpha,t)$ be as defined in (2.2), and let $e_{0}(t,r)$ be the proportional exponential function. Then the proportional integral is defined as

19 $$ _{P}\mathbf{I}^{\alpha }\phi (t)= \int _{a}^{t}e_{0}(t,r)\phi (r) \,d_{\alpha }r, \quad d_{\alpha }r=\frac{1}{\kappa _{0}(\alpha,r)}\,dr. $$

### Lemma 2.5

([15])

*Let* $\alpha \le 0\le 1$, *let* $\Theta:[r,t]\rightarrow \mathbb{R}$ *be a continuous function*, *and let* $\kappa _{1}(\alpha,t)$ *and* $\kappa _{0}(\alpha,t)$ *be defined as in* (2.2). *Then the proportional derivative* ${}_{P}\mathbf{D}^{\alpha }$ *has some desired properties*:

(i) ${}_{P}\mathbf{D}^{\alpha }[c_{1}{\phi (t)}+c_{2}{\varphi (t)}]=c_{1}{_{P} \mathbf{D}^{\alpha }}[\phi (t)]+c_{2}{_{P}\mathbf{D}^{\alpha }}[\varphi (t)]$ *for all* $c_{1},c_{2}\in \mathbb{R}$.
(ii) ${}_{P}\mathbf{D}^{\alpha }c=c\kappa _{1}(\alpha,\cdot )$ *for all* $c\in \mathbb{R}$.
(iii) ${}_{P}\mathbf{D}^{\alpha }[\phi (t)\varphi (t)]=\phi (t){_{P}\mathbf{D}^{\alpha }}[\varphi (t)]+\varphi (t){_{P}\mathbf{D}^{\alpha }}[\phi (t)]- \phi (t)\varphi (t)\kappa _{1}(\alpha,\cdot )$.
(iv) ${}_{P}\mathbf{D}^{\alpha } [\frac{\phi (t)}{\varphi (t)} ]= \frac{\varphi (t){_{P}\mathbf{D}^{\alpha }}[\phi (t)]-\phi (t){_{P}\mathbf{D}^{\alpha }}[\varphi (t)]}{\varphi ^{2}(t)}+ \frac{\phi (t)}{\varphi (t)}\kappa _{1}(\alpha,\cdot )$.
(v) *For* $r\in \mathbb{R}$ *and* $0<\alpha \le 1$,
  20 $$ _{P}\mathbf{D}^{\alpha }\bigl[e_{\Theta }(t,r)\bigr]=\Theta (t)e_{\Theta }(t,r), $$
  *where* $e_{\Theta }(t,r)$ *is the proportional exponential function*.
(vi) *Let* $0<\alpha \le 1$, *and let* $e_{0}(t,r)$ *be the proportional exponential function*. *Then*
  21 $$ _{P}\mathbf{D}^{\alpha } \biggl[ \int _{a}^{t}{e_{0}(t,r)\phi (r) \,d_{\alpha }r} \biggr]=\phi (t),\quad d_{\alpha }r= \frac{1}{\kappa _{0}(\alpha,r)}\,dr. $$

### Definition 2.6

([15])

Let $y_{1},y_{2}:[t_{0},\infty )$ be *α*-differentiable functions on $[t_{0},\infty )$. Then the proportional Wronskian (p-Wronskian) of $y_{1}(t)$ and $y_{2}(t)$ is presented by

22 $$ W_{p}(y_{1},y_{2})= \begin{vmatrix} y_{1}(t) & y_{2}(t) \\ _{P}\mathbf{D}^{\alpha }y_{1}(t) & _{P} \mathbf{D}^{\alpha }y_{1}(t) \end{vmatrix} . $$

### Definition 2.7

([8])

The truncated *M*-derivative of $f:[0,\infty )\rightarrow \mathbb{R}$ for $0<\alpha <1$ is

23 $$ _{M}\mathbf{D}_{M}^{\alpha,\beta }f(t)=\lim _{\varepsilon \rightarrow 0} \frac{f(t E_{\beta }(\varepsilon t^{-\alpha }))-f(t)}{\varepsilon },\quad t>0, $$

where $E_{\beta }(\cdot )$, $\beta >0$, is the truncated Mittag-Leffler function.

### Definition 2.8

([6])

Assuming that $f:[0,\infty )\rightarrow \mathbb{R}$, the conformable derivative is defined by

24 $$ _{C}\mathbf{D}_{\alpha }f(t)=\lim_{\varepsilon \rightarrow 0} \frac{f(t+\varepsilon t^{1-\alpha })-f(t)}{\varepsilon } $$

for $t>0$ and $0<\alpha <1$.

## Some methods via proportional derivative

### Proportional variation-of-parameters method

Here we show the proportional variation-of-parameters method for a constant- or variable-coefficient linear differential equation of order *nα*. The main purpose is to find a particular solution to the equation

25 $$ L_{\alpha }[y](t)=g(t), $$

where

26 $$ L_{\alpha }[y]={_{P}\mathbf{D}^{(n)\alpha }}y+r_{1}{_{P} \mathbf{D}^{(n-1) \alpha }}y+\cdots+{r_{n}}y, $$

where $0<\alpha <1$, ${}_{P}\mathbf{D}^{(n)\alpha } = \underbrace{_{P}\mathbf{D}^{\alpha }{_{P}\mathbf{D}^{\alpha }}\cdots _{P}\mathbf{D}^{\alpha }}_{ \text{n-times}}$, and ${r_{1}},\ldots,{r_{n}}$ and *g* are continuous functions on an interval $(a,b)$. This method requires that the fundamental solution set $\{y_{1},\ldots,y_{n}\}$ for the corresponding homogeneous equation $L_{\alpha }[y](x)=0$ is already known as follows:

27 $$ y_{h}(t)=c_{1}y_{1}(t)+ \cdots+c_{n}y_{n}(t), $$

where $c_{1},\ldots,c_{n}$ are arbitrary constants, and the function *y* is *nα* times differentiable. To find a particular solution, we replace $c_{1},\ldots,c_{n}$ in Eq. (27) by functions $\gamma _{1}(t),\ldots,\gamma _{n}(t)$. So, in proportional variation-of-parameters method, we suppose that there is a particular solution to (25) of the form

28 $$ y_{p}(x)=\gamma _{1}(t)y_{1}(t)+\cdots+\gamma _{n}(t)y_{n}(t), $$

and then the functions $\gamma _{1},\ldots,\gamma _{n}$ are determined.

Particularly, let us consider proportional nonhomogeneous linear differential equation of order 2*α*

29 $$ _{P}\mathbf{D}^{\alpha }{_{P} \mathbf{D}^{\alpha }}y(t)+a{_{P}\mathbf{D}^{ \alpha }}y(t)+by(t)=g(t), $$

where $a,b$ are constants or functions. Let $y_{1}(t)$ and $y_{2}(t)$ be two linearly independent solutions for

30 $$ _{P}\mathbf{D}^{\alpha }{_{P} \mathbf{D}^{\alpha }}y(t)+a{_{P}\mathbf{D}^{ \alpha }}y(t)+by(t)=0. $$

Hence we seek a solution of equation (29) of the form

31 $$ y_{p}(t)=\gamma _{1}(t)y_{1}(t)+ \gamma _{2}(t)y_{2}(t). $$

After that, by taking the proportional derivative of (31) we have

32 $$\begin{aligned} _{P}\mathbf{D}^{\alpha }y_{p}(t)={}&{_{P} \mathbf{D}^{\alpha }}\bigl[\gamma _{1}(t)y_{1}(t)+ \gamma _{2}(t)y_{2}(t)\bigr] \\ ={}& \kappa _{1}(\alpha,t)\bigl[\gamma _{1}(t)y_{1}(t)+ \gamma _{2}(t)y_{2}(t)\bigr]+{ \kappa _{0}}( \alpha,t)\bigl[\gamma _{1}(t)y_{1}(t)+\gamma _{2}(t)y_{2}(t)\bigr]' \\ ={}& \kappa _{1}(\alpha,t)\gamma _{1}(t)y_{1}(t)+ \kappa _{1}(\alpha,t) \gamma _{2}(t)y_{2}(t) \\ &{}+ \kappa _{0}(\alpha,t)\gamma '_{1}(t)y_{1}(t)+ \kappa _{0}(\alpha,t) \gamma _{1}(t)y'_{1}(t) \\ &{}+ \kappa _{0}(\alpha,t)\gamma '_{2}(t)y_{2}(t)+ \kappa _{0}(\alpha,t) \gamma _{2}(t)y'_{2}(t). \end{aligned}$$

To get rid of the second-order derivatives of the functions $\gamma _{1}$, $\gamma _{2}$ in ${_{P}\mathbf{D}^{\alpha }}{_{P}\mathbf{D}^{\alpha }}y_{p}(t)$, from now on we make the following assumption:

33 $$ \kappa _{0}(\alpha,t)\gamma '_{1}(t)y_{1}(t)+ \kappa _{0}(\alpha,t) \gamma '_{2}(t)y_{2}(t)=0. $$

Calculating the proportional derivative of the function $y_{p}(t)$ once again, we get

34 $$\begin{aligned} {_{P}\mathbf{D}^{\alpha }} {_{P}\mathbf{D}^{\alpha }}y_{p}(t)={}&{_{P} \mathbf{D}^{\alpha }}\bigl[\kappa _{1}(\alpha,t)\gamma _{1}(t)y_{1}(t)+ \kappa _{1}(\alpha,t)\gamma _{2}(t)y_{2}(t) \end{aligned}$$

35 $$\begin{aligned} &{}+ \kappa _{0}(\alpha,t)\gamma _{1}(t)y'_{1}(t)+ \kappa _{0}(\alpha,t) \gamma _{2}(t)y'_{2}(t) \bigr], \\ {_{P}\mathbf{D}^{\alpha }} {_{P}\mathbf{D}^{\alpha }}y_{p}(t)={}& \kappa _{1}( \alpha,t)\bigl[\kappa _{1}(\alpha,t)\gamma _{1}(t)y_{1}(t)+\kappa _{1}( \alpha,t)\gamma _{2}(t)y_{2}(t) \end{aligned}$$

36 $$\begin{aligned} &{}+ \kappa _{0}(\alpha,t)\gamma _{1}(t)y'_{1}(t)+ \kappa _{0}(\alpha,t) \gamma _{2}(t)y'_{2}(t) \bigr] \\ &{}+ \kappa _{0}(\alpha,t)\bigl[\kappa _{1}( \alpha,t)\gamma _{1}(t)y_{1}(t)+ \kappa _{1}( \alpha,t)\gamma _{2}(t)y_{2}(t) \\ &{}+ \kappa _{0}(\alpha,t)\gamma _{1}(t)y'_{1}(t)+ \kappa _{0}(\alpha,t) \gamma _{2}(t)y'_{2}(t) \bigr]', \\ {_{P}\mathbf{D}^{\alpha }} {_{P}\mathbf{D}^{\alpha }}y_{p}(t)={}& \kappa ^{2}_{1}( \alpha,t)\gamma _{1}(t)y_{1}(t)+ \kappa ^{2}_{1}(\alpha,t)\gamma _{2}(t)y_{2}(t)+ \kappa _{0}(\alpha,t)\kappa _{1}(\alpha,t)\gamma _{1}(t)y'_{1}(t) \\ &{}+ \kappa _{0}(\alpha,t)\kappa _{1}(\alpha,t) \gamma _{2}(t)y'_{2}(t)+ \kappa _{0}(\alpha,t)\kappa '_{1}(\alpha,t)\gamma _{1}(t)y_{1}(t) \\ &{}+ \kappa _{0}(\alpha,t)\kappa _{1}(\alpha,t) \gamma '_{1}(t)y_{1}(t)+ \kappa _{0}(\alpha,t)\kappa _{1}(\alpha,t)\gamma _{1}(t)y'_{1}(t) \\ &{}+ \kappa _{0}(\alpha,t)\kappa '_{1}( \alpha,t)\gamma _{2}(t)y_{2}(t)+ \kappa _{0}( \alpha,t)\kappa _{1}(\alpha,t)\gamma '_{2}(t)y_{2}(t) \\ &{}+ \kappa _{0}(\alpha,t)\kappa _{1}(\alpha,t) \gamma _{2}(t)y'_{2}(t)+ \kappa _{0}(\alpha,t)\kappa '_{0}(\alpha,t)\gamma _{1}(t)y'_{1}(t) \\ &{}+ \kappa ^{2}_{0}(\alpha,t)\gamma '_{1}(t)y'_{1}(t)+\kappa ^{2}_{0}( \alpha,t)\gamma _{1}(t)y''_{1}(t) \\ &{}+ \kappa _{0}(\alpha,t)\kappa '_{0}( \alpha,t)\gamma _{2}(t)y'_{2}(t)+ \kappa ^{2}_{0}(\alpha,t)\gamma '_{2}(t)y'_{2}(t) \\ &{}+ \kappa ^{2}_{0}(\alpha,t)\gamma _{2}(t)y''(t). \end{aligned}$$

Substituting (32) and (36) into (29) yields

37 $$\begin{aligned} & \kappa ^{2}_{1}(\alpha,t)\gamma _{1}(t)y_{1}(t)+ \kappa ^{2}_{1}( \alpha,t)\gamma _{2}(t)y_{2}(t)+ \kappa _{0}(\alpha,t)\kappa _{1}( \alpha,t)\gamma _{1}(t)y'_{1}(t) \\ &\quad{}+ \kappa _{0}(\alpha,t)\kappa _{1}(\alpha,t) \gamma _{2}(t)y'_{2}(t)+ \kappa _{0}(\alpha,t)\kappa '_{1}(\alpha,t)\gamma _{1}(t)y_{1}(t) \\ &\quad{}+ \kappa _{0}(\alpha,t)\kappa _{1}(\alpha,t) \gamma '_{1}(t)y_{1}(t)+ \kappa _{0}(\alpha,t)\kappa _{1}(\alpha,t)\gamma _{1}(t)y'_{1}(t) \\ &\quad{}+ \kappa _{0}(\alpha,t)\kappa '_{1}( \alpha,t)\gamma _{2}(t)y_{2}(t)+ \kappa _{0}( \alpha,t)\kappa _{1}(\alpha,t)\gamma '_{2}(t)y_{2}(t) \\ &\quad{}+ \kappa _{0}(\alpha,t)\kappa _{1}(\alpha,t) \gamma _{2}(t)y'_{2}(t)+ \kappa _{0}(\alpha,t)\kappa '_{0}(\alpha,t)\gamma _{1}(t)y'_{1}(t) \\ &\quad{}+ \kappa ^{2}_{0}(\alpha,t)\gamma '_{1}(t)y'_{1}(t)+\kappa ^{2}_{0}( \alpha,t)\gamma _{1}(t)y''_{1}(t)+ \kappa _{0}(\alpha,t)\kappa '_{0}( \alpha,t) \gamma _{2}(t)y'_{2}(t) \\ &\quad{}+ \kappa ^{2}_{0}(\alpha,t)\gamma '_{2}(t)y'_{2}(t)+\kappa ^{2}_{0}( \alpha,t)\gamma _{2}(t)y''_{2}(t)+a \kappa _{1}(\alpha,t)\gamma _{1}(t)y_{1}(t) \\ &\quad{}+ a\kappa _{1}(\alpha,t)\gamma _{2}(t)y_{2}(t)+a \kappa _{0}( \alpha,t)\gamma _{1}(t)y'_{1}(t)+a \kappa _{0}(\alpha,t)\gamma _{2}(t)y'_{2}(t) \\ &\quad{}+ b\gamma _{1}(t)y_{1}(t)+b\gamma _{2}(t)y_{2}(t)=g(t). \end{aligned}$$

Then we can get

38 $$ \kappa ^{2}_{0}(\alpha,t)\gamma '_{1}(t)y'_{1}(t)+\kappa ^{2}_{0}( \alpha,t)\gamma '_{2}(t)y'_{2}(t)=g(t). $$

We next utilize assumption (33) and equation (38) to find the functions $\gamma _{1}(t)$ and $\gamma _{2}(t)$. For this purpose, we write

39 $$ \begin{pmatrix} y_{1}(t)&y_{2}(t) \\ y'_{1}(t)&y'_{2}(t) \end{pmatrix} \begin{pmatrix} \gamma '_{1}(t) \\ \gamma '_{2}(t) \end{pmatrix}= \begin{pmatrix} 0 \\ \frac{g(t)}{\kappa ^{2}_{0}(\alpha,t)} \end{pmatrix} $$

and thus obtain

40 $$ \begin{pmatrix} \gamma '_{1}(t) \\ \gamma '_{2}(t) \end{pmatrix}=\frac{1}{W(y_{1},y_{2})(t)} \begin{pmatrix} -y_{2}(t)\frac{g(t)}{\kappa ^{2}_{0}(\alpha,t)} \\ y_{1}(t)\frac{g(t)}{\kappa ^{2}_{0}(\alpha,t)} \end{pmatrix}. $$

So we can readily reach the formulas

41 $$ \gamma '_{1}(t)= \frac{-y_{2}(t)g(t)}{\kappa ^{2}_{0}(\alpha,t)W(y_{1},y_{2})(t)}\quad \text{and} \quad\gamma '_{2}(t)= \frac{y_{1}(t)g(t)}{\kappa ^{2}_{0}(\alpha,t)W(y_{1},y_{2})(t)}. $$

By choosing $\kappa _{1}(\alpha,t)=1-\alpha $ and $\kappa _{0}(\alpha,t)=\alpha $, which we may in fact assume, the proportional variation-of-parameters method can be presented with similar calculations, and so we also have

42 $$ \gamma '_{1}(t)=\frac{-y_{2}(t)g(t)}{\alpha ^{2} {W(y_{1},y_{2})(t)}}\quad \text{and}\quad \gamma '_{2}(t)= \frac{y_{1}(t)g(t)}{\alpha ^{2} {W(y_{1},y_{2})(t)}}. $$

After integrating the functions $\gamma '_{1}(t)$ and $\gamma '_{2}(t)$, we get the stated result.

#### Application 3.1

Let us consider a mass-spring system driven by a external force $g(t)$ at time *t*. The mass of spring system is $m>0$, the damping constant is $2b>0$, the spring constant is $k>0$, and the displacement from the equilibrium of the mass-spring system at time *t* is denoted by $y(t)$. So the motion is governed by

43 $$ m{_{P}\mathbf{D}^{\alpha }} {_{P} \mathbf{D}^{\alpha }}y(t)+2b{_{P}\mathbf{D}^{\alpha }}y(t)+ky(t)=g(t),\quad t\in [t_{0},\infty ). $$

To solve this equation, we use the proportional variation-of-parameters method. Therefore to reach the general solution of (43), we first need the corresponding auxiliary equation

44 $$ m\lambda ^{2}+2b\lambda +k=0. $$

We have three cases for finding the solution of the homogeneous part of equation (43):

(i) If $mk< b^{2}$, then we have
  45 $$ y_{h}(t)=c_{1}{e_{\frac{-b+\sqrt{b^{2}-mk}}{m}}(t,0)}+c_{2}{e_{ \frac{-b-\sqrt{b^{2}-mk}}{m}}(t,0)}. $$
(ii) If $mk=b^{2}$, then we have
  46 $$ y_{h}(t)=c_{1}{e_{-b/a}(t,0)}+c_{2}{e_{-b/a}(t,0)} \int _{0}^{t} \,d_{\alpha }s. $$
(iii) If $mk>b^{2}$, then we have
  47 $$\begin{aligned} y_{h}(t)={}&c_{1}{e_{-b/m}(t,0)\cos \biggl( \int _{0}^{t} \frac{\sqrt{mk-b^{2}}}{m}\,d_{\alpha }s \biggr)} \\ &{}+c_{2}{e_{-b/m}(t,0)\sin \biggl( \int _{0}^{t} \frac{\sqrt{mk-b^{2}}}{m}\,d_{\alpha }s \biggr)}. \end{aligned}$$

Let us begin with case (iii) and presume that

48 $$\begin{aligned} y_{p}(t)={}&\gamma _{1}(t){e_{-b/m}(t,0) \cos \biggl( \int _{0}^{t} \frac{\sqrt{mk-b^{2}}}{m}\,d_{\alpha }s \biggr)} \\ &{}+\gamma _{2}(t){e_{-b/m}(t,0) \cos \biggl( \int _{0}^{t} \frac{\sqrt{mk-b^{2}}}{m}\,d_{\alpha }s \biggr)}. \end{aligned}$$

The *p*-Wronskian can be computed by

49 $$ W_{p}= \begin{vmatrix} e^{\frac{-b-(1-\alpha )m}{m\alpha }t}\cos \biggl( \frac{\sqrt{mk-b^{2}}}{m\alpha }t \biggr) & e^{ \frac{-b-(1-\alpha )m}{m\alpha }t}\sin \biggl( \frac{\sqrt{mk-b^{2}}}{m\alpha }t \biggr) \\ _{P}\mathbf{D}^{\alpha }{ \biggl[e^{\frac{-b-(1-\alpha )m}{m\alpha }t} \cos \biggl( \frac{\sqrt{mk-b^{2}}}{m\alpha }t \biggr) \biggr]} & _{P} \mathbf{D}^{\alpha }{ \biggl[e^{\frac{-b-(1-\alpha )m}{m\alpha }t}\sin \biggl(\frac{\sqrt{mk-b^{2}}}{m\alpha }t \biggr) \biggr]} \end{vmatrix} , $$

where

50 $$\begin{aligned} {_{P}\mathbf{D}^{\alpha }} \biggl[e^{\frac{-b-(1-\alpha )m}{m\alpha }t} \cos \biggl( \frac{\sqrt{mk-b^{2}}}{m\alpha }t \biggr) \biggr]={}& e^{ \frac{-b-(1-\alpha )m}{m\alpha }t} \biggl[ \biggl(- \frac{b}{m} \biggr) \cos \biggl(\frac{\sqrt{mk-b^{2}}}{m} \biggr) \\ &{}- \biggl(\frac{\sqrt{mk-b^{2}}}{m} \biggr)\sin \biggl( \frac{\sqrt{mk-b^{2}}}{m} \biggr) \biggr] \end{aligned}$$

and

51 $$\begin{aligned} {_{P}\mathbf{D}^{\alpha }} \biggl[e^{\frac{-b-(1-\alpha )m}{m\alpha }t} \sin \biggl( \frac{\sqrt{mk-b^{2}}}{m\alpha }t \biggr) \biggr]={}& e^{ \frac{-b-(1-\alpha )m}{m\alpha }t} \biggl[ \biggl(- \frac{b}{m} \biggr) \sin \biggl(\frac{\sqrt{mk-b^{2}}}{m} \biggr) \\ &{}+ \biggl(\frac{\sqrt{mk-b^{2}}}{m} \biggr)\cos \biggl( \frac{\sqrt{mk-b^{2}}}{m} \biggr) \biggr]. \end{aligned}$$

Hence we have

52 $$ W_{p}= \biggl(\frac{\sqrt{mk-b^{2}}}{m} \biggr)e^{ \frac{2(-b-m+\alpha m)}{m\alpha }t}. $$

Using formulas (42), we get

53 $$ \gamma '_{1}(t)= \frac{-e^{\frac{-b-(1-\alpha )m}{m\alpha }t}\sin (\frac{\sqrt{mk-b^{2}}}{m\alpha }t )g(t)}{\alpha ^{2} W_{p}} $$

and

54 $$ \gamma '_{2}(t)= \frac{e^{\frac{-b-(1-\alpha )m}{m\alpha }t}\cos (\frac{\sqrt{mk-b^{2}}}{m\alpha }t )g(t)}{\alpha ^{2} W_{p}}. $$

So, taking integrals of (53) and (54), we find the functions $\gamma _{1}(t)$ and $\gamma _{2}(t)$. Lastly, by inserting the functions $\gamma _{1}(t)$ and $\gamma _{2}(t)$ into the (48) we get the desired result. Note that similar calculations can be readily done for cases (i) and (ii).

### Proportional Laplace transform

In this portion, we provide a detailed exposition of proportional derivative and the corresponding Laplace transform. We examine the proportional Laplace transform (LT-p) method to be utilized in solving initial value problems. This method is a substantial transformation used in mathematics, physics, engineering, and other applied sciences. Hence, as an alternative to the usual Laplace transform, we present its generalized version to obtain novel solutions containing arbitrary order *α*. As we mentioned in Sect. 2, a particular case of proportional derivative of order *α* is given by

55 $$ _{P}\mathbf{D}^{\alpha }{\phi (t)}=(1-\alpha )\phi (t)+\alpha \phi '(t), $$

where $\phi '(t)$ is the traditional derivative of the function $\phi (t)$. If we apply the usual Laplace transform to both sides of (55), then using the equality $\mathcal{L}\{\phi '(t)\}=s\mathcal{L}\{\phi (t)\}-\phi (0)$, we get

56 $$ \mathcal{L}\bigl\{ _{P}\mathbf{D}^{\alpha }\phi (t)\bigr\} =(\alpha s+1-\alpha ) \mathcal{L}\bigl\{ \phi (t)\bigr\} -\alpha \phi (0). $$

Taking advantage of the above *α*-order derivative, we first compute ${}_{P}\mathbf{D}^{(n)\alpha }\phi (t)$ to derive its Laplace transform. To this end, for $n=2$, we have

57 $$ _{P}\mathbf{D}^{\alpha }\bigl[_{P} \mathbf{D}^{\alpha }\phi (t)\bigr]={_{P} \mathbf{D}^{(2)\alpha } \phi (t)}=\alpha ^{2}{\phi ''(t)}+2\alpha (1- \alpha )\phi '(t)+(1-\alpha )^{2}{\phi (t)}, $$

and taking the Laplace transform of (57), we get

58 $$ \mathcal{L}\bigl\{ _{P}\mathbf{D}^{(2)\alpha }\phi (t)\bigr\} =( \alpha s+1-\alpha )^{2} \mathcal{L}\bigl\{ \phi (t)\bigr\} -\alpha \bigl[ \alpha s+2(1-\alpha )\bigr]\phi (0)- \alpha ^{2}{\phi '(0)}. $$

Also, for $n=3$, we have

59 $$ _{P}\mathbf{D}^{(3)\alpha }\phi (t)=\alpha ^{3}{\phi '''(t)}+3\alpha ^{2}(1- \alpha )\phi ''(t)+3\alpha (1-\alpha )^{2}{\phi '(t)}+(1-\alpha )^{3}{ \phi (t)}, $$

and by applying the Laplace transform to (59) we obtain

60 $$\begin{aligned} \mathcal{L}\bigl\{ _{P}\mathbf{D}^{(3)\alpha }\phi (t)\bigr\} ={}&( \alpha s+1- \alpha )^{3}{\mathcal{L}\bigl\{ \phi (t)\bigr\} }-\alpha \bigl[\alpha ^{2} s^{2}+3 \alpha s(1-\alpha )+3(1-\alpha )^{2}\bigr]\phi (0) \\ &{}- \alpha ^{2}\bigl[\alpha s+3(1-\alpha )\bigr]\phi '(0)-\alpha ^{3}{\phi ''(0)}. \end{aligned}$$

After carrying out same process *n* times, we readily find

61 $$\begin{aligned} _{P}\mathbf{D}^{(n)\alpha }\phi (t)={}& \begin{pmatrix} n \\ 0 \end{pmatrix}\alpha ^{n}{\phi ^{(n)}(t)}+ \begin{pmatrix} n \\ 1 \end{pmatrix}\alpha ^{n-1}(1-\alpha )\phi ^{(n-1)}(t) \\ &{}+ \begin{pmatrix} n \\ 2 \end{pmatrix}\alpha ^{n-2}(1-\alpha )^{2}{\phi ^{(n-2)}(t)}+\cdots+ \begin{pmatrix} n \\ r \end{pmatrix}\alpha ^{n-r}(1-\alpha )^{r}{\phi ^{(n-r)}(t)} \\ &{}+\cdots + \begin{pmatrix} n \\ n \end{pmatrix}(1-\alpha )^{n}{ \phi (t)}, \end{aligned}$$

where ${}_{P}\mathbf{D}^{(n)\alpha } = \underbrace{_{P}\mathbf{D}^{\alpha }{_{P}\mathbf{D}^{\alpha }}\cdots _{P}\mathbf{D}^{\alpha }}_{ \text{$n$ times}}$, and by taking the Laplace transform of (61) we have

62 $$\begin{aligned} \mathcal{L}\bigl\{ _{P}\mathbf{D}^{(n)\alpha } \phi (t)\bigr\} ={}&(\alpha s+1- \alpha )^{n}{\mathcal{L}\bigl\{ \phi (t) \bigr\} }-\alpha \left[ \begin{pmatrix} n \\ 0 \end{pmatrix}(\alpha s)^{n-1}+ \begin{pmatrix} n \\ 1 \end{pmatrix}(\alpha s)^{n-2}(1- \alpha )\right. \\ &{}+ \begin{pmatrix} n \\ 2 \end{pmatrix}(\alpha s)^{n-3}(1-\alpha )^{2}+\cdots + \begin{pmatrix} n \\ r \end{pmatrix}(\alpha s)^{n-r-1}(1-\alpha )^{r}+\cdots \\ &{}+ \left. \begin{pmatrix} n \\ {n-1} \end{pmatrix}(1-\alpha )^{n-1} \right]\phi (0)-\alpha ^{2} \left[ \begin{pmatrix} n \\ 0 \end{pmatrix}(\alpha s)^{n-2}+ \begin{pmatrix} n \\ 1 \end{pmatrix}(\alpha s)^{n-3}(1-\alpha ) \right. \\ &{}+ \begin{pmatrix} n \\ 2 \end{pmatrix}(\alpha s)^{n-4}(1-\alpha )^{2}+\cdots + \begin{pmatrix} n \\ r \end{pmatrix}(\alpha s)^{n-r-2}(1-\alpha )^{r}+\cdots \\ &{}+\left. \begin{pmatrix} n \\ {n-2} \end{pmatrix}(1-\alpha )^{n-2} \right]\phi '(0)-\alpha ^{3} \left[ \begin{pmatrix} n \\ 0 \end{pmatrix}(\alpha s)^{n-3}+ \begin{pmatrix} n \\ 1 \end{pmatrix}(\alpha s)^{n-4}(1-\alpha )\right. \\ &{}+ \begin{pmatrix} n \\ 2\ \end{pmatrix}(\alpha s)^{n-5}(1-\alpha )^{2}+\cdots + \begin{pmatrix} n \\ r \end{pmatrix}(\alpha s)^{n-r-3}(1-\alpha )^{r}+\cdots \\ &{}+ \left. \begin{pmatrix} n \\ {n-3} \end{pmatrix}(1-\alpha )^{n-3} \right]\phi ''(0)-\cdots - \alpha ^{n}{\phi ^{(n-1)}(0)}, \end{aligned}$$

where $\alpha \in (0,1]$, $\phi \in C^{n-1}[0,\infty )$ defined in [11] is a piecewise continuous function having exponential order in the interval $0\le t\le N$, $N>0$, and $\mathcal{L}\{\phi (t)\}=\int _{0}^{\infty }e^{-st}\phi (t)\,dt$ is the classical Laplace transform.

It is worth pointing out that for $\alpha =1$ in (62), we get the usual Laplace transform of *n*th-order derivative of the function $\phi (t)$.

## Differential equations in clinical medicine by means of local derivatives

In this section, we use different types of local derivatives for some crucial differential equations in clinical medicine. We observe the mechanical action performed by the ventilator used for critically ill patients. To this end, from now on we make the following assumptions:

- The length of each breath is denoted by $t_{b}$, which is determined by the clinician. Each breath is assumed to consist of two stages, inspiration and expiration, and $t_{j}$ stands for the inspiratory time. In addition, the lung is modeled by a single compartment.
- We denote by $P_{d}$ the pressure of the ventilator to the air-way of patient during expiration.
- We considered the pressure balance at the airway as follows:
  63 $$ P_{l}+P_{k}+P_{m}=P_{aw}, $$
  where $P_{l}$ is the airway-resistance drop, the lung elastic pressure is denoted by $P_{k}$, and the residual pressure is denoted by $P_{m}$. Note that $P_{aw}=P_{d}$ during inspiration and $P_{aw}=0$ during expiration.

### Clinical medicine model via proportional derivative

Considering the pressure equation (63) and all the assumptions above, the instantaneous volume in a lung by means of local proportional derivative is presented by

64 $$\begin{aligned} & R \bigl[_{P}\mathbf{D}^{\alpha }{ \mathcal{V}_{i}(t)} \bigr]+ \biggl( \frac{1}{C} \biggr) \mathcal{V}_{i}(t)+P_{m}=P_{d}, \quad0 \le t\le t_{j}, \end{aligned}$$

65 $$\begin{aligned} & R \bigl[_{P}\mathbf{D}^{\alpha }{ \mathcal{V}_{e}(t)} \bigr]+ \biggl( \frac{1}{C} \biggr) \mathcal{V}_{e}(t)+{P_{m}}=0,\quad t_{j} \le t\le t_{b}, \end{aligned}$$

66 $$\begin{aligned} & \mathcal{V}_{i}(0)=\mathcal{V}_{e}(t_{b})=0, \end{aligned}$$

67 $$\begin{aligned} &\mathcal{V}_{i}(t_{j})= \mathcal{V}_{e}(t_{j})=\mathcal{V}_{T}, \end{aligned}$$

where $\mathcal{V}_{i}(t)$ is the lung volume during inspiration, and $\mathcal{V}_{e}(t)$ is the lung volume during expiration. Also, *R* is a proportionality constant, which is the same for both inspiration and expiration, and *C* is a constant called the compliance of the lung. It should be mentioned that $P_{m}$ can be determined from the condition $\mathcal{V}_{e}(t_{b})=0$.

Let us first solve equation (64) by means of LT-p introduced in Sect. 3. If we take the LT-p of equation (64), then by using the initial condition (66),we get

68 $$\begin{aligned} &R\mathcal{L}\bigl\{ _{P}\mathbf{D}^{\alpha }{\mathcal{V}_{i}(t)} \bigr\} + \biggl( \frac{1}{C} \biggr)\mathcal{L}\bigl\{ \mathcal{V}_{i}(t) \bigr\} +=\mathcal{L}\{P_{d}-P_{m} \}, \end{aligned}$$

69 $$\begin{aligned} &(\alpha s+1-\alpha )\mathcal{L}\bigl\{ \mathcal{V}_{i}(t)\bigr\} - \alpha \mathcal{V}_{i}(0)+ \biggl(\frac{1}{CR} \biggr)\mathcal{L} \bigl\{ \mathcal{V}_{i}(t)\bigr\} =\mathcal{L} \biggl\{ \frac{P_{d}-P_{m}}{R} \biggr\} , \end{aligned}$$

70 $$\begin{aligned} & \mathcal{L}\bigl\{ \mathcal{V}_{i}(t)\bigr\} = \frac{C(P_{d}-P_{m})}{s+C(R+\alpha R(s-1))s}. \end{aligned}$$

Applying the inverse LT to (70), we obtain

71 $$ \mathcal{V}_{i}(t)=C(P_{d}-P_{m}) \biggl( \frac{1}{1+CR-\alpha CR}- \frac{e^{-\frac{(1+CR-\alpha CR)t}{\alpha CR}}}{1+CR-\alpha CR} \biggr). $$

In a similar way, solving equation (65) under condition (66) with the help of LT-p, we have the solution

72 $$ \mathcal{V}_{e}(t)=-CP_{m} \biggl(\frac{1}{1+CR-\alpha CR}- \frac{e^{-\frac{(1+CR-\alpha CR)(t-t_{b})}{\alpha CR}}}{1+CR-\alpha CR} \biggr). $$

On the other hand, let us solve equation (64) under condition (67). After taking the LT-p of (64), we follow the steps

73 $$\begin{aligned} &R\mathcal{L}\bigl\{ _{P}\mathbf{D}^{\alpha }\mathcal{V}_{i}(t) \bigr\} +\frac{1}{C} \mathcal{L}\bigl\{ \mathcal{V}_{i}(t)\bigr\} = \mathcal{L}\{P_{d}-P_{m}\}, \end{aligned}$$

74 $$\begin{aligned} &\mathcal{L}\bigl\{ \mathcal{V}_{i}(t)\bigr\} = \frac{C(P_{d}-P_{m}+\alpha Rs\mathcal{V}_{T})}{s+C(R+\alpha R(s-1))s}, \end{aligned}$$

and applying the inverse LT, we get

75 $$\begin{aligned} \mathcal{V}_{i}(t)={}& C \biggl(\frac{P_{d}-P_{m}}{-1-CR+\alpha CR} \biggr) \\ &{}+ \frac{e^{-\frac{(1+CR-\alpha CR)(t-t_{j})}{\alpha CR}}(\alpha CRP_{d}-\alpha CRP_{m}-\alpha R\mathcal{V}_{T}-\alpha CR^{2} \mathcal{V}_{T}+\alpha ^{2} CR^{2} \mathcal{V}_{T})}{\alpha R(-1-CR+\alpha CR)}. \end{aligned}$$

Similarly, solving equation (65) under condition (67) by means of LT-p, we readily obtain the solution

76 $$ \mathcal{V}_{e}(t)= \frac{e^{-\frac{(1+CR-\alpha CR)(t-t_{j})}{\alpha CR}} (-CP_{m}+Ce^{\frac{(1+CR-\alpha CR)(t-t_{j})}{\alpha CR}}P_{m}-\mathcal{V}_{T}-CR\mathcal{V}_{T}+\alpha CR\mathcal{V}_{T} )}{-1-CR+\alpha CR}. $$

### Clinical medicine model via truncated *M*-derivative

Under the above-stated assumptions, the instantaneous volume in a lung by means of truncated *M*-derivative can be expressed by

77 $$\begin{aligned} & R \bigl[_{M}\mathbf{D}^{\alpha,\beta } \mathcal{V}_{i}(t) \bigr]+ \biggl(\frac{1}{C} \biggr) \mathcal{V}_{i}(t)+P_{m}=P_{d},\quad 0\le t\le t_{j}, \end{aligned}$$

78 $$\begin{aligned} & R \bigl[_{M}\mathbf{D}^{\alpha,\beta } \mathcal{V}_{e}(t) \bigr]+ \biggl(\frac{1}{C} \biggr) \mathcal{V}_{e}(t)+P_{m}=0, \quad t_{j} \le t\le t_{b}, \end{aligned}$$

79 $$\begin{aligned} & \mathcal{V}_{i}(0)=\mathcal{V}_{e}(t_{b})=0, \end{aligned}$$

80 $$\begin{aligned} &\mathcal{V}_{i}(t_{j})= \mathcal{V}_{e}(t_{j})=\mathcal{V}_{T}. \end{aligned}$$

Solving equation (77) with the condition $\mathcal{V}_{i}(0)=0$ in (79) by means of LT, we can write

81 $$\begin{aligned} &R\mathcal{L}_{\alpha,\beta }\bigl\{ _{M}\mathbf{D}^{\alpha,\beta } \mathcal{V}_{i}(t)\bigr\} + \biggl(\frac{1}{C} \biggr) \mathcal{L}_{\alpha, \beta }\bigl\{ \mathcal{V}_{i}(t)\bigr\} = \mathcal{L}_{\alpha,\beta } \{ P_{d}-P_{m} \}, \end{aligned}$$

82 $$\begin{aligned} &s\mathcal{L}_{\alpha,\beta }\bigl\{ \mathcal{V}_{i}(t)\bigr\} - \mathcal{V}_{i}(0)+ \biggl(\frac{1}{CR} \biggr) \mathcal{L}_{\alpha,\beta }\bigl\{ \mathcal{V}_{i}(t) \bigr\} = \frac{P_{d}-P_{m}}{sR}, \end{aligned}$$

83 $$\begin{aligned} & \mathcal{L}_{\alpha,\beta }\bigl\{ \mathcal{V}_{i}(t) \bigr\} = \frac{C(P_{d}-P_{m})}{s+CRs^{2}}, \end{aligned}$$

and taking the inverse Laplace transform of (83), we obtain the solution

84 $$ \mathcal{V}_{i}(t)=C(P_{d}-P_{m}) \bigl(1-e^{ \frac{-\Gamma (\beta +1)}{CR}\frac{t^{\alpha }}{\alpha }} \bigr). $$

Similarly, solving equation (78) with the condition $\mathcal{V}_{e}(t_{b})=0$ as in (79) via LT, we get

85 $$ \mathcal{V}_{e}(t)=CP_{m} \bigl(-1+e^{\frac{-\Gamma (\beta +1)}{CR} \frac{(t-t_{b})^{\alpha }}{\alpha }} \bigr). $$

Also, let us solve equation (77) under the condition $\mathcal{V}_{i}(t_{j})=\mathcal{V}_{T}$ in (80) by using the LT as follows:

86 $$\begin{aligned} &R\mathcal{L}_{\alpha,\beta }\bigl\{ _{M}\mathbf{D}^{\alpha,\beta } \mathcal{V}_{i}(t)\bigr\} +\frac{1}{C}\mathcal{L}_{\alpha,\beta } \bigl\{ \mathcal{V}_{i}(t)\bigr\} =\mathcal{L}_{\alpha,\beta } \{P_{d}-P_{m}\}, \end{aligned}$$

87 $$\begin{aligned} &s\mathcal{L}_{\alpha,\beta }\bigl\{ \mathcal{V}_{i}(t)\bigr\} - \mathcal{V}_{i}(t_{j})+ \frac{1}{CR} \mathcal{L}_{\alpha,\beta }\bigl\{ \mathcal{V}_{i}(t)\bigr\} = \frac{P_{d}-P_{m}}{Rs}, \end{aligned}$$

88 $$\begin{aligned} &\mathcal{L}_{\alpha,\beta }\bigl\{ \mathcal{V}_{i}(t) \bigr\} = \frac{C(P_{d}-P_{m}+Rs\mathcal{V}_{T})}{s(1+CRs)}, \end{aligned}$$

and by applying the inverse Laplace transform to equation (88) we readily obtain

89 $$ \mathcal{V}_{i}(t)=C(P_{d}-P_{m})+e^{\frac{-\Gamma (\beta +1)}{CR} \frac{(t-t_{j})^{\alpha }}{\alpha }} \bigl[C(P_{d}-P_{m})+\mathcal{V}_{T} \bigr]. $$

In a similar manner, taking the LT of equation (78) with the condition $\mathcal{V}_{e}(t_{j})=\mathcal{V}_{T}$ in (80), we get the solution

90 $$ \mathcal{V}_{e}(t)=-CP_{m}+e^{\frac{-\Gamma (\beta +1)}{CR} \frac{(t-t_{j})^{\alpha }}{\alpha }}(CP_{m}+ \mathcal{V}_{T}). $$

### Clinical medicine model via conformable derivative

Under the essential assumptions stated above, the instantaneous volume in a lung by means of conformable derivative can be given by

91 $$\begin{aligned} & R \bigl[_{C}\mathbf{D}^{\alpha } \mathcal{V}_{i}(t) \bigr]+ \biggl( \frac{1}{C} \biggr) \mathcal{V}_{i}(t)+P_{m}=P_{d},\quad 0 \le t\le t_{j}, \end{aligned}$$

92 $$\begin{aligned} &R \bigl[_{C}\mathbf{D}^{\alpha } \mathcal{V}_{e}(t) \bigr]+ \biggl( \frac{1}{C} \biggr) \mathcal{V}_{e}(t)+P_{m}=0,\quad t_{j} \le t\le t_{b}, \end{aligned}$$

93 $$\begin{aligned} &\mathcal{V}_{i}(0)=\mathcal{V}_{e}(t_{b})=0, \end{aligned}$$

94 $$\begin{aligned} & \mathcal{V}_{i}(t_{j})= \mathcal{V}_{e}(t_{j})=\mathcal{V}_{T}. \end{aligned}$$

Solving equation (91) under condition (93) with the help of LT, we have

95 $$\begin{aligned} &R\mathcal{L}_{\alpha }\bigl\{ _{C}\mathbf{D}^{\alpha } \mathcal{V}_{i}(t)\bigr\} + \biggl(\frac{1}{C} \biggr) \mathcal{L}_{\alpha }\bigl\{ \mathcal{V}_{i}(t)\bigr\} = \mathcal{L}_{\alpha }\{P_{d}-P_{m}\}, \end{aligned}$$

96 $$\begin{aligned} &s\mathcal{L}_{\alpha }\bigl\{ \mathcal{V}_{i}(t)\bigr\} - \mathcal{V}_{i}(0)+ \biggl( \frac{1}{CR} \biggr) \mathcal{L}_{\alpha }\bigl\{ \mathcal{V}_{i}(t)\bigr\} = \frac{P_{d}-P_{m}}{sR}, \end{aligned}$$

97 $$\begin{aligned} & \mathcal{L}_{\alpha }\bigl\{ \mathcal{V}_{i}(t) \bigr\} = \frac{C(P_{d}-P_{m})}{s+CRs^{2}}, \end{aligned}$$

and if we apply the inverse LT to both sides of equation (97), we get the solution

98 $$ \mathcal{V}_{i}(t)=C(P_{d}-P_{m}) \bigl(1-e^{- \frac{t^{\alpha }}{\alpha CR}} \bigr). $$

Also, for equation (92) with condition (93), we can present the solution

99 $$ \mathcal{V}_{e}(t)=CP_{m} \bigl(-1+e^{- \frac{(t-t_{b})^{\alpha }}{\alpha CR}} \bigr). $$

On the other hand, let us give the solution by means of LT for equation (91) with condition (94):

100 $$ \mathcal{V}_{i}(t)=C(P_{d}-P_{m})+e^{- \frac{(t-t_{j})^{\alpha }}{\alpha CR}} \bigl[C(P_{d}-P_{m})+\mathcal{V}_{T} \bigr]. $$

Similarly, the solution of equation (92) under condition (94) is

101 $$ \mathcal{V}_{e}(t)=-CP_{m}+e^{-\frac{(t-t_{j})^{\alpha }}{\alpha CR}}(CP_{m}+ \mathcal{V}_{T}). $$

## Discussions and conclusions

**Figure 1:**
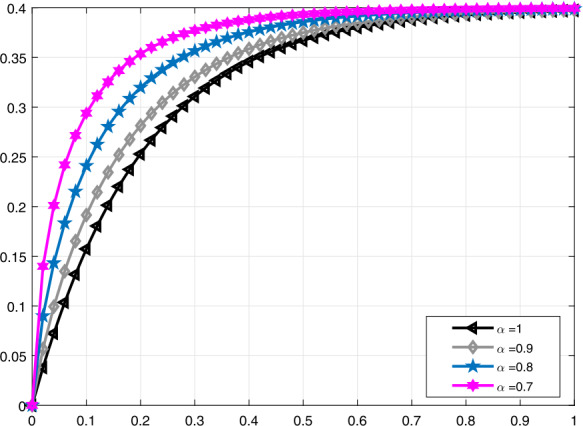
Comparative analysis with truncated *M*-derivative for $\mathcal{V}_{i}(t)$, $\beta =0.8$

**Figure 2:**
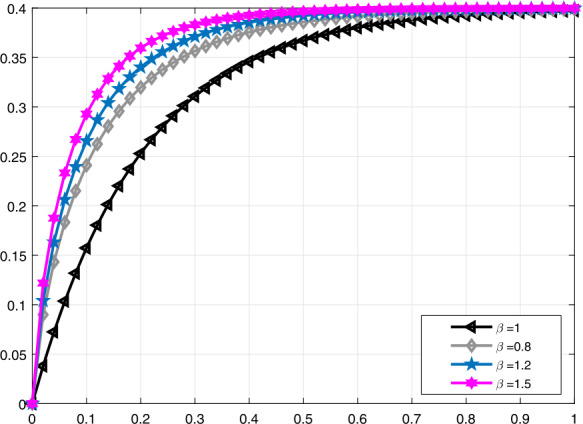
Comparative analysis with truncated *M*-derivative for $\mathcal{V}_{i}(t)$, $\alpha =0.8$
- In Fig. 3, a comparison is made for $\mathcal{V}_{i}(t)$ when $\alpha =1,0.95,0.82,0.68$, and in Fig. 4, it is made for $\alpha =1,0.95,0.9,0.85$. Moreover, in Fig. 5 the solution curves of $\mathcal{V}_{i}(t)$ are shown by means of proportional derivative for $\alpha =1,0.65,0.45,0.25$ and in Fig. 6 for $\alpha =1,0.9,0.8,0.7$.

We list some important conclusions and discussion on our results:

- This study has provided a natural and intrinsic characterization of a significant application in medicine describing the instantaneous volume in a lung under by means of the proportional derivative defined by using the PD controller, *M*-derivative, including the truncated Mittag-Leffler function, and conformable derivative.
- Besides examining the model stated, we have offered alternative solution methods, which can be used in other crucial problems in nature. These methods, proportional variation of parameters and proportional Laplace transform, have been introduced through the proportional derivative, which is a generalized version of the conformable derivative.
- It is worth mentioning the main reason for utilizing proportional derivatives. Local derivatives of noninteger order have more advantages than their counterparts as their are defined for $\alpha \in [0,1]$ and $t\in \mathbb{R}$, which makes possible to get the identity operator for $\alpha =1$, whereas conformable and modified conformable derivatives do not satisfy this important property.
- From the two useful methods we provided we have chosen an appropriate one to obtain solutions for the clinical medicine model we examined. Moreover, in addition to the proportional derivatives, we have also taken advantage of two other derivatives for clearly observing the instantaneous volume of the lung.
- In addition to being an important supportive treatment, mechanical ventilation may also create some risk factors on patients. Hence patients on a ventilator are carefully monitored by the health team. The possibility of lung collapse due to getting full of air makes it necessary to observe the instantaneous volume of the lung as in this study. To perform this observation in detail, we separately show the solution curves of $\mathcal{V}_{i}(t)$ and $\mathcal{V}_{e}(t)$.
- In Fig. 1, we have carried out a comparison in terms of truncated *M*-derivative for the function $\mathcal{V}_{i}(t)$ standing for the lung volume during inspiration when $\alpha =1,0.9,0.8,0.7$ and $\beta =0.8$. This allows us to see the increase in the volume of the lung at different times and when it is stable. Also, a similar approach was made for Fig. 2, that is, the volume of lung $\mathcal{V}_{i}(t)$ was plotted for $\beta =1,0.8,1.2,1.5$ and $\alpha =0.8$ to observe the effect of *α* and *β* on solution curves.

**Figure 3:**
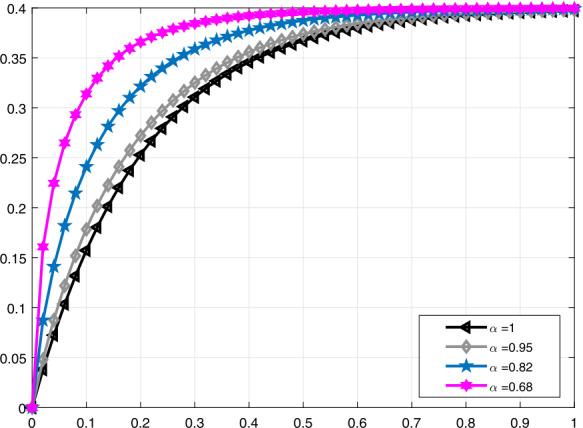
Comparative analysis with conformable derivative for $\mathcal{V}_{i}(t)$

**Figure 4:**
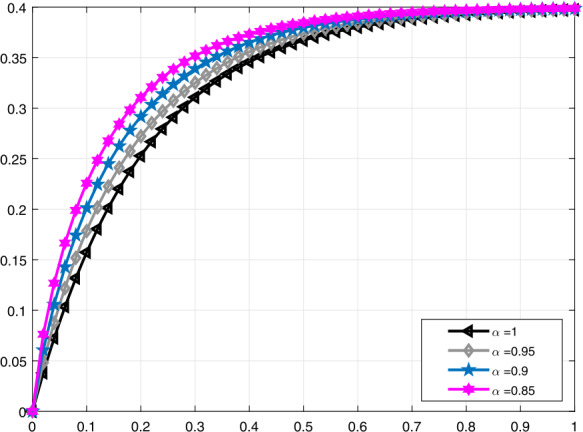
Comparative analysis with conformable derivative for $\mathcal{V}_{i}(t)$

**Figure 5:**
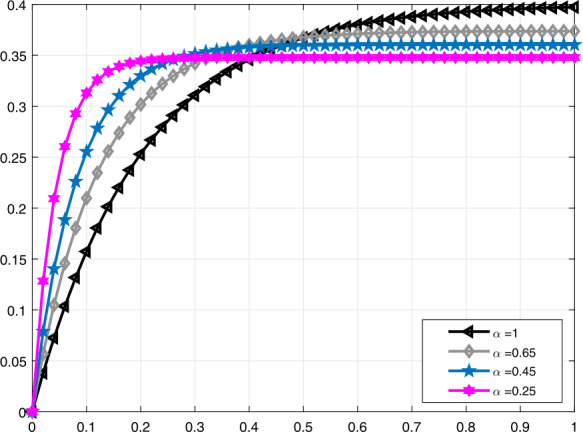
Comparative analysis with proportional derivative for $\mathcal{V}_{i}(t)$

**Figure 6:**
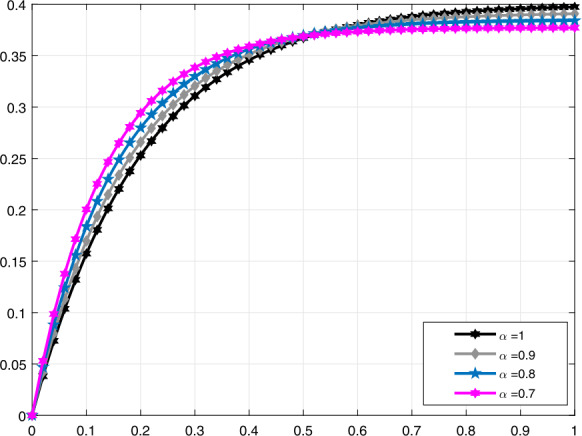
Comparative analysis with proportional derivative for $\mathcal{V}_{i}(t)$
- In Figs. 7 and 8, we compare the proportional derivative, truncated *M*-derivative, and conformable derivative with the traditional one for the function $\mathcal{V}_{i}(t)$ when $\alpha =0.75$, $\beta =0.5$ and $\alpha =0.9$, $\beta =0.8$, respectively. We can clearly seen that the proportional derivative tends to be close to the classical derivative faster than the truncated *M*-derivative and conformable derivative.

**Figure 7:**
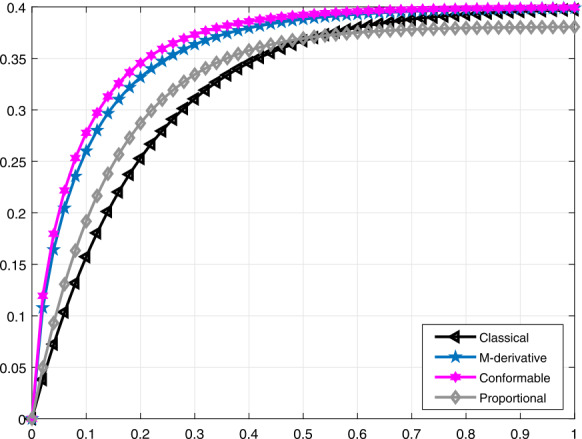
Comparative analysis when $\alpha =0.75$ and $\beta =0.5$ for $\mathcal{V}_{i}(t)$

**Figure 8:**
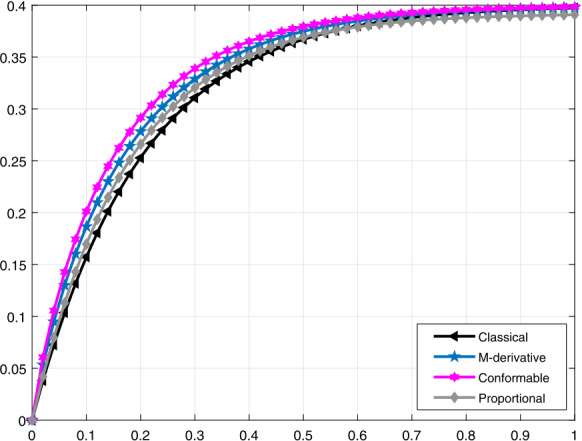
Comparative analysis when $\alpha =0.9$ and $\beta =0.8$ for $\mathcal{V}_{i}(t)$
- Lastly, in Figs. 9–12, similar comparisons for the function $\mathcal{V}_{e}(t)$ are presented, which enables us to observe the decrease in volume of the lung during expiration at different times *t* for different values of *α* and *β*.

**Figure 9:**
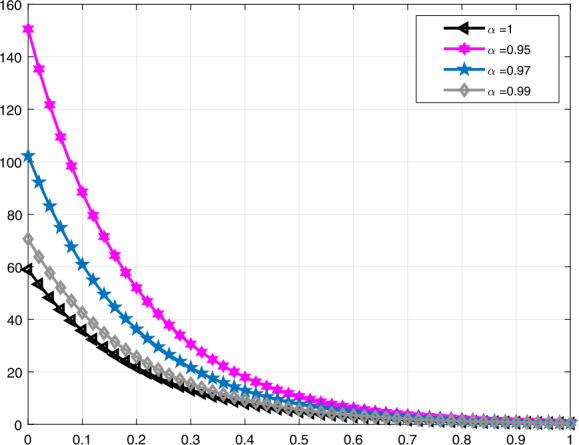
Comparative analysis with proportional derivative for $\mathcal{V}_{e}(t)$

**Figure 10:**
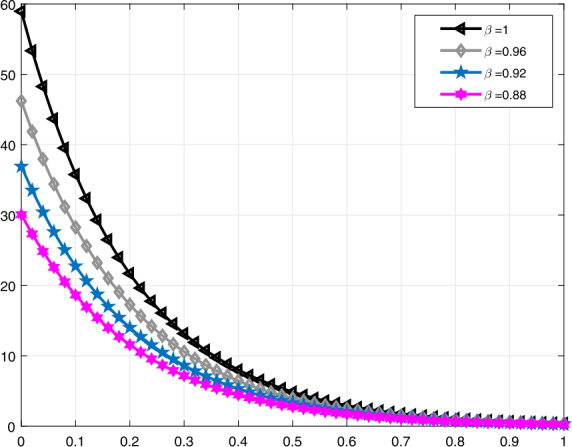
Comparative analysis with truncated *M*-derivative for $\mathcal{V}_{e}(t)$, $\alpha =1$

**Figure 11:**
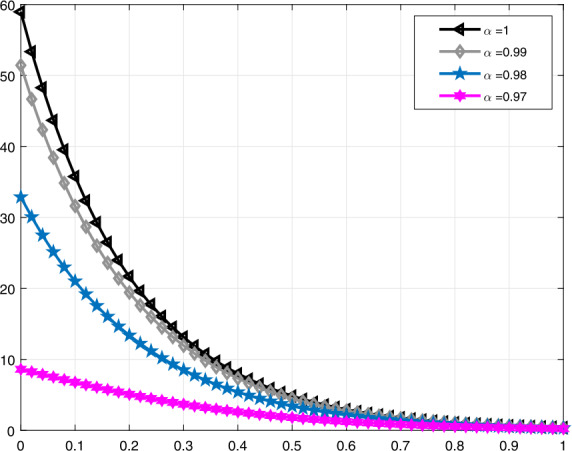
Comparative analysis with conformable derivative for $\mathcal{V}_{e}(t)$

**Figure 12:**
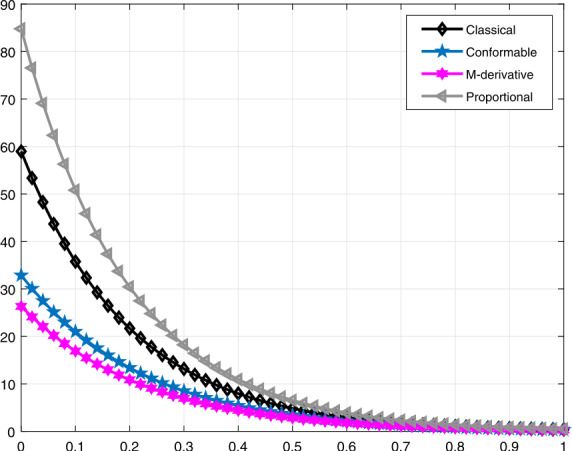
Comparative analysis when $\alpha =0.98$ and $\beta =0.96$ for $\mathcal{V}_{e}(t)$
- It should be noted that all graphs are plotted for $R=10$ cm (H_2_O)/L/sec, $C=0.02\text{ L}/\text{cm}$(H_2_O), $P_{d}=20$ cm (H_2_O), $t_{j}=1$ sec, and $t_{b}=3$ sec. Additionally, note that all solutions obtained by the proportional derivative, truncated *M*-derivative, and conformable derivative correspond to the classical solution of the model analyzed when $\alpha =1$.

## Data Availability

Data sharing not applicable to this paper as no datasets were generated or analyzed during the current study.
